# Chest Magnetic Resonance Imaging Decreases Inter-observer Variability of Gross Target Volume for Lung Tumors

**DOI:** 10.3389/fonc.2019.00690

**Published:** 2019-08-13

**Authors:** Laurent Basson, Hajer Jarraya, Alexandre Escande, Abel Cordoba, Rayyan Daghistani, David Pasquier, Thomas Lacornerie, Eric Lartigau, Xavier Mirabel

**Affiliations:** ^1^Universitary Radiation Oncology Department, Oscar Lambret Comprehensive Cancer Center, Lille, France; ^2^University of Lille, Lille, France; ^3^Medical Imaging Department, Oscar Lambret Comprehensive Cancer Center, Lille, France; ^4^Department of Medical Physics, Oscar Lambret Comprehensive Cancer Center, Lille, France

**Keywords:** lung cancer, GTV, chest MRI, inter-observer variability, delineation

## Abstract

**Purpose:** PET/CT is a standard medical imaging used in the delineation of gross tumor volume (GTV) in case of radiation therapy for lung tumors. However, PET/CT could present some limitations such as resolution and standardized uptake value threshold. Moreover, chest MRI has shown good potential in diagnosis for thoracic oncology. Therefore, we investigated the influence of chest MRI on inter-observer variability of GTV delineation.

**Methods and Materials:** Five observers contoured the GTV on CT for 14 poorly defined lung tumors during three contouring phases based on true daily clinical routine and acquisition: CT phase, with only CT images; PET phase, with PET/CT; and MRI phase, with both PET/CT and MRI. Observers waited at least 1 week between each phases to decrease memory bias. Contours were compared using descriptive statistics of volume, coefficient of variation (COV), and Dice similarity coefficient (DSC).

**Results:** MRI phase volumes (median 4.8 cm^3^) were significantly smaller than PET phase volumes (median 6.4 cm^3^, *p* = 0.015), but not different from CT phase volumes (median 5.7 cm^3^, *p* = 0.30). The mean COV was improved for the MRI phase (0.38) compared to the CT (0.58, *p* = 0.024) and PET (0.53, *p* = 0.060) phases. The mean DSC of the MRI phase (0.67) was superior to those of the CT and PET phases (0.56 and 0.60, respectively; *p* < 0.001 for both).

**Conclusions:** The addition of chest MRI seems to decrease inter-observer variability of GTV delineation for poorly defined lung tumors compared to PET/CT alone and should be explored in further prospective studies.

## Introduction

Target volume delineation is a key step in radiotherapy planning. It is widely considered as one of the main sources of error in radiotherapy. Many studies have been conducted to reduce variability, whether by applying guidelines, teaching procedures or using automated contours, or by using an additional imaging examination (1, 2).

Important discordance has been observed in the determination of gross tumor volume (GTV) in lung cancer (3). For this site, positron emission tomography/computed tomography (PET/CT) has shown a decrease in inter-observer variability. It is thus considered the standard and is used routinely (4–8). However, PET/CT presents some limitations as an aid for contouring (9). The resolution is ~4–8 mm, which is particularly unsatisfactory for stereotactic radiotherapy, in which the need for accuracy is of utmost importance (8). Image acquisition is done over several minutes, which results in a volume between GTV, mid-position, and internal target volume (10). Moreover, there is still no identification of a standardized uptake value (SUV) threshold (8, 10, 11). Thus, it is important to improve PET/CT characteristics and to improve contouring accuracy.

Magnetic resonance imaging (MRI) plays an important role in different aspects of the treatment of many tumors, especially in improving delineation (12). The use of MRI in thoracic oncology has also recently increased due to improved image quality of chest MRI. Substantial advancements in MRI technology have made it possible to overcome some common problems, such as respiratory motion and low proton density of the lung tissue, which have represented an obstacle for the use of this imaging exam in the past. Compared to PET/CT, chest MRI demonstrated good potential in discriminating benign and malignant tumors (13, 14), predicting tumor aggressiveness (13, 15), assessing the nodal staging (16–18), and predicting response to treatment (19, 20). In radiotherapy, chest MRI provides unique anatomical imaging and evaluation of tumor motion (21). Nevertheless, at the time the study was conducted, no studies had been published to specifically evaluate inter-observer variability in tumor delineation using chest MRI (21).

Thus, the aim of this study was to assess the effect of MRI, when combined with CT and PET/CT, on GTV delineation for lung tumors.

## Materials and Methods

### Clinical Cases

Fourteen anonymous and previously treated clinical cases were used. They were treated between November 2013 and October 2015. All consecutive patients who had pulmonary radiotherapy treatment following a chest MRI performed in our center were selected. There was no case of mediastinal node. MRI was performed to aid in the delineation of tumors which were considered to be poorly defined on CT by the radiation oncologist, mainly because of associated atelectasis (*n* = 8). The other reasons of poor definition and the case characteristics are presented in Table 1. Radiotherapy was delivered, either by stereotactic radiotherapy or by conformal radiotherapy. All patients had been informed about the opportunity of using the recorded data in later studies following French law on informatics and liberty and the study was approved by the Center Oscar Lambret ethics committee. Written consent is not needed because it is retrospective, monocentric study, without any intervention.

**Table 1:** Cases characteristics.

**Case**	**Target**	**Histology**	**Size max (mm)**	**SUV max**	**Difficulty**	**Radiation technique**	**Difficulty mean NS**
1	Primitive	SCC	37	16.6	Atelectasis	SR	4.0
2	Relapse	SCC	19	8.9	RP	SR	7.2
3	Primitive	SCC	55	17.5	Atelectasis	CR	8.0
4	Primitive	ADK	22	6.5	Atelectasis	SR	4.4
5	Primitive	SCC	20	5.4	Atelectasis	SR	6.4
6	Relapse	SCC	27	7.4	Atelectasis	SR	5.6
7	Primitive	LCC	47	9.8	Peri-hilar	SR	4.4
8	Primitive	SCC	19	7.0	Atelectasis	SR	9.6
9	Relapse	Unknown	14	6.1	RP	SR	2.6
10	Primitive	SCC	13	3.2	Occult on CT	SR	9.6
11	Relapse	SCLL	26	9.4	Peri-hilar + CICE	SR	4.0
12	Primitive	ADK	41	31.8	Atelectasis	CR	7.4
13	Metastasis	Rectal ADK	19	20.1	Peri-hilar + CICE	SR	7.2
14	Primitive	ADK	60	8.6	Atelectasis	CR	5.6

*SUV, Standardized Uptake Value; NS, Numeric Scale; SCC, Squamous Cell Carcinoma; ADK, Adenocarcinoma; LCC, Large-cell Carcinoma; SCLL, Small Cell Lung Cancer; RP, Radiation Pneumonitis; CICE, Contraindication for Contrast Enhancement; SR, Stereotactic Radiotherapy; CR, Conformal Radiotherapy*.

### Observers

Five physicians from our institution, four radiation oncologists (three seniors and one resident with 25, 19, 12, and 4 years of experience, respectively) and one radiologist (one resident with 4 years of experience), were asked to delineate. They all received a short training session on chest MRI by a dedicated radiologist responsible for performing chest MRI in our center.

### Medical Imaging Protocols

CT scan was performed on a Toshiba Aquilion LB CT scanner. The contrast agent was 100 mL of iodine 350 mg/mL (Optiject®, Guerbet, Villepinte, France). There was no contrast enhancement for two cases: one allergy to iodinated contrast and one chronic renal failure. For stereotactic radiotherapy, free breathing, 4D (four respiratory phases), and breath hold acquisition were used, and the slice thickness was 1 mm. For conformal radiotherapy, free breathing acquisition was used, and the slice thickness was 2 mm.

PET/CT was done for a diagnostic purpose on a General Electric Discovery PET/CT scanner (11 cases), Philips Gemini TF16 PET/CT scanner (two cases), or Siemens mCT Flow PET/CT scanner (one case). Patients underwent a whole-body ^18^F-FDG PET-CT with image acquisition for ~1 h after weight-adjusted ^18^F-FDG intravenous injection (average 238 MBq, range 139–368). The PET slice thicknesses were 3.27, 4.00, and 2.03 mm, according to the machine.

Chest MRI was performed for delineation purposes on a General Electric SIGNA MR750 3.0 Tesla scanner. The contrast media used was 12–20 mL gadoteric acid (Dotarem®, Guerbet, Villepinte, France). The protocol included the following sequences: axial and coronal T2 with fat saturation weighted images (propeller), axial T1 3D (Lava Flex)-weighted images before, and after contrast enhancement, coronal T1 3D (Lava Flex)-weighted images at a delayed phase. For breathing motion, T2 used respiratory trigger, and T1 sequences used end inspiration breath hold for 8–20 s. The slice thickness was 2.6 mm for T1 and 3 mm for T2.

For CT, the position of patients was supine, with two arms above their head (11 cases) or two arms along the body (three cases). For PET/CT, it was supine with two arms above their head. For MRI, it was supine with two arms along the body. PET/CT and MRI were not performed in the radiation treatment position.

### Delineation

All delineations were manually performed on the treatment planning system Oncentra MasterPlan® (Elekta AB, Stockholm, Sweden). For the three phases of delineation, observers delineated a single GTV on the contrast-enhanced acquisition, if available. If not available, they performed the delineation on the sharpest acquisition of the CT scan. Neither the Internal Target Volume (ITV), nor the Planning Target Volume (PTV) were assessed.

For the first phase of the study, the CT phase, observers used only the CT scans performed during treatment planning. To approach “real life” conditions (2), all pretreatment documents and images were available except PET/CT and MRI images and reports. The delineation had to be done slice by slice, and checked on the lung window (−600/1600 HU), mediastinal window (+20/400 HU), and in the three anatomic views (axial, coronal, sagittal). Observers were free to vary the window level according to their convenience.

For the second phase, the PET phase, a new delineation was performed on CT scan using PET/CT images as an aid for delineation with the PET/CT report available. There was no SUV threshold recommended following the 2014 International Atomic Energy Agency consensus report (8).

For the third phase, the MRI phase, a final delineation with the help of the three imaging modalities, CT scan, PET/CT, and MRI, was performed. Both weighted T1 and T2 were primarily used by the observers. The MRI report was available for this phase.

Image fusion by a rigid registration centered on the tumor was done for PET/CT and MRI (T1-weighted sequence) with CT and validated by final clinical visual examination. These registrations were considered as an aid for the observers, but the final contour had to be done on CT. Each delineation session was performed over a minimum time interval of 1 week. The help of a nuclear physician or radiologist was not allowed. Observers were blinded to their previous contours and to other physicians' contours. After the CT phase, physicians were asked to assess the difficulty of delineation of each case on a numeric scale from 0 (very easy) to 10 (very difficult). Following this assessment, two groups were created: the seven cases with the highest mean numeric scale were classified in the “more difficult” group, and the seven other cases were classified in the “less difficult” group.

### Statistical Analysis

Descriptive statistics of the five volumes for each phase of every case are presented with the mean and median volume. To assess inter-observer variability, the first index used was the coefficient of variation (COV). The COV is defined as the ratio of the standard deviation to the mean volume. One COV was calculated by phase for every case. The more homogeneous the volume size, the more is the decrease in the COV value.

(1) $$\begin{array}{r} \text{COV}=\frac{\sigma }{\mu } \end{array}$$

The Dice similarity coefficient (DSC) was the second index used. The DSC quantifies the overlap between two volumes. The DSC corresponds to the ratio of twice the intersection of two volumes divided by the sum of the two volumes, where *A* and *B* are the two volumes to be compared. A value of one indicates a total concordance between the two volumes, whereas a value of 0 indicates the absence of any overlap between the two volumes.

(2) $$\begin{array}{r} \text{DSC}=\frac{2\left(A\cap B\right)}{A+B} \end{array}$$

For each phase in every case, 10 DSC values were calculated to correspond to the different combinations of two contours from the five observers. Case 10 had only six DSC values because one of the observers did not delineate the case since he treated it previously. An additional analysis was performed according to the group difficulty.

A global DSC, per phase of every case, was calculated, where *A, B, C, D*, and *E* are the contours of the five different observers for a similar phase, such as:

(3) $$\begin{array}{r} \text{Global DSC}=\frac{5\left(A\cap B\cap C\cap D\cap E\right)}{A+B+C+D+E} \end{array}$$

Datasets were imported to the Artiview® platform for contour analysis. Phase comparisons of contour volumes, COV, and DSC means were performed using two-sided paired *t*-tests or Wilcoxon paired tests, if appropriate. Comparisons between the more and less difficult group were done by two-sided non-paired *t*-test. A *p*-value < 0.05 was considered to be significant. *R* version 3.4.3 was used for descriptive and analytic statistics.

## Results

### Volumes and COV

Volume characteristics are presented in Table 2. The mean of all volumes in cm^3^ was 29.3 for the CT phase, 31.7 for the PET phase, and 24.7 for the MRI phase. The median of all volumes in cm^3^ was 5.7 for the CT phase, 6.4 for the PET phase, and 4.8 for the MRI phase. MRI phase volumes were significantly smaller compared to PET phase volumes (*p* = 0.015), but not statistically different from CT phase volumes (*p* = 0.30).

**Table 2:** Volume characteristics per phase of delineation.

**Case**		**Mean (cm3)**			**Median (cm3)**			**COV**		
		**CT**	**PET**	**MRI**	**CT**	**PET**	**MRI**	**CT**	**PET**	**MRI**
LDG	1	17.6	19.3	16.8	16.5	19.9	15.4	0.18	0.26	0.28
	4	3.6	2.4	3.0	3.7	2.3	2.8	0.13	0.45	0.25
	6	15.7	8.4	5.1	15.6	6.6	4.7	0.56	0.68	0.42
	7	35.5	31.0	29.3	31.2	33.3	29.7	0.33	0.45	0.15
	9	4.4	4.5	3.8	4.1	4.3	3.5	0.25	0.25	0.15
	11	6.9	6.7	6.5	6.4	5.8	7.4	0.34	0.34	0.24
	14	170.7	214.2	165.3	148.6	193.3	141.9	0.34	0.36	0.44
MDG	2	2.6	2.8	3.8	2.1	2.3	4.2	0.59	0.65	0.40
	3	99.9	75.0	42.1	26.2	35.2	42.5	1.22	1.10	0.16
	5	5.6	4.3	3.9	5.6	4.4	4.3	0.72	0.26	0.27
	8	4.3	4.0	2.9	1.8	5.0	3.6	0.97	0.77	0.65
	10	0.8	1.5	1.5	0.5	1.3	0.9	1.31	0.98	1.16
	12	34.0	59.7	54.8	34.8	63.9	59.4	0.52	0.25	0.35
	13	2.6	4.2	2.4	1.8	2.7	2.4	0.65	0.61	0.40
Mean								0.58	0.53	0.38

*CT, Computed Tomography; PET, Positron Emission Tomography; MRI, Magnetic Resonance Imaging; COV, Coefficient of Variation; LDG, Less Difficult group; MDG, More Difficult Group*.

**Figure 1:**
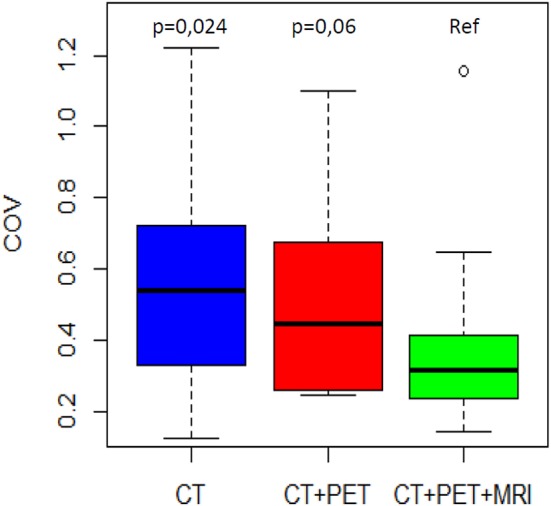
COV distribution by phase of delineation. The MRI phase is compared to the CT phase and the PET/CT phase.

The mean COVs were 0.58, 0.53, and 0.38 for the CT, PET, and MRI phases, respectively (Table 2). Variability in volume dimensions for the MRI phase was lower than that of the CT phase (*p* = 0.024) but not significant compared to the PET phase (*p* = 0.060; Figure 1). There was no difference in COV between the CT and PET phases (*p* = 0.37). The assessment of the delineation difficulty is presented in Table 1. COV values were higher in the more difficult group compared to the less difficult group (mean 0.67 vs. 0.32, respectively; *p* < 0.001). For the more difficult group, the difference between phases was more significant: mean COV of 0.85, 0.66, and 0.48 for the CT, PET, and MRI phases, respectively (CT vs. PET *p* = 0.047, CT vs. MRI *p* = 0.016, PET vs. MRI *p* = 0.30). In contrast, for the less difficult group, the differences between phases were lower: mean COV of 0.30, 0.40, and 0.27 for the CT, PET, and MRI phases, respectively (CT vs. PET *p* = 0.047, CT vs. MRI *p* = 0.47, PET vs. MRI *p* = 0.08).

### DSC

**Figure 2:**
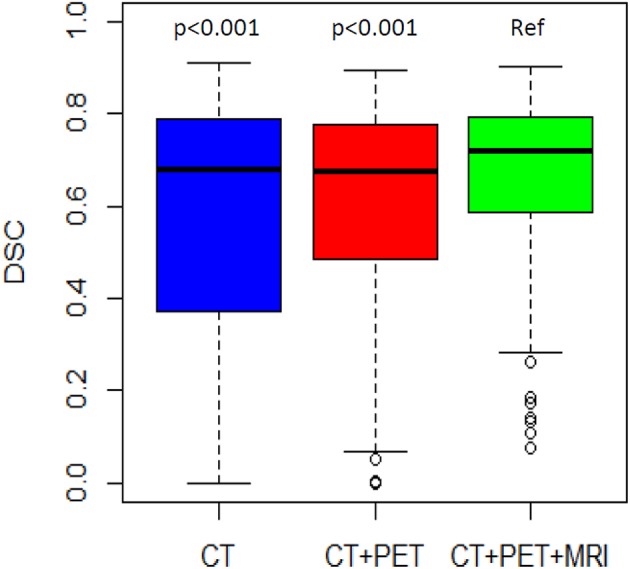
DSC distribution by phase of delineation. The MRI phase is compared to the CT phase and the PET/CT phase.

DSC distributions are shown in Figure 2. The mean DSCs were 0.56, 0.60, and 0.67 for the CT, PET, and MRI phases, respectively. The MRI phase was statistically superior to the CT and PET phases (*p* < 0.001 for both). Moreover, the PET phase did not reach a statistical superiority over the CT phase for mean DSC (*p* = 0.056). The mean values of the 14 global DSC values were 0.33, 0.34, and 0.41 for the CT, PET, and MRI phases, respectively.

**Figure 3:**
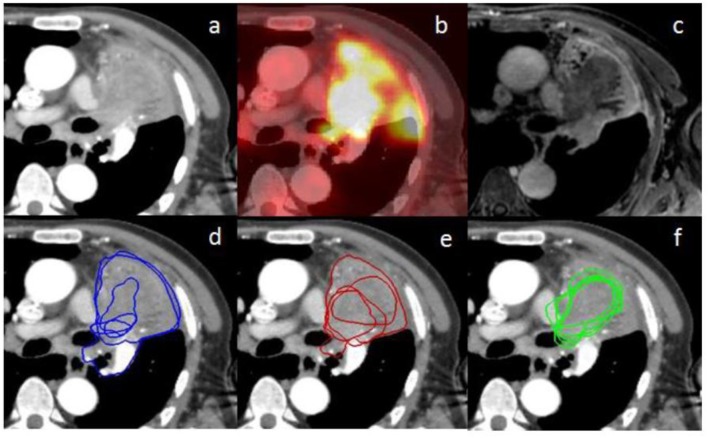
**(a)** represents an axial planning CT scan on the mediastinal window (+20/400 HU); **(b)** represents the fused PET scan corresponding to the planning CT scan **(a)** on rigid registration; **(c)** represents the MRI weighted T1 Lava Flex Water sequence at a delayed phase (8 min) corresponding to the planning CT scan **(a)** on rigid registration; **(d–f)** represent the contours from the five different observers, respectively, at the CT phase, PET phase, and MRI phase. There are only four visible contours for the PET phase since one of the volumes is outside, up to the CT scan. Inter-observer variability is lower for the MRI phase compared to the other phases.

The mean DSC was lower in the more difficult group compared to the less difficult group (0.47 vs. 0.74, respectively; *p* < 0.001). For the less difficult group, there was not much difference between the mean DSCs which were 0.75, 0.72, and 0.75 for the three phases, respectively (Table 3). However, for the more difficult group (example in Figure 3), the mean DSCs were lower, and the difference between phases was more important with mean DSCs of 0.36, 0.47, and 0.58 for the CT, PET, and MRI phases, respectively (*p* < 0.001 for the three possible comparisons).

**Table 3:** Mean DSC per phase of delineation.

**Case**		**Mean DSC**		
		**CT**	**PET**	**MRI**
LDG	1	0.86	0.81	0.80
	4	0.74	0.67	0.71
	6	0.56	0.57	0.63
	7	0.78	0.66	0.86
	9	0.81	0.82	0.82
	11	0.75	0.74	0.74
	14	0.75	0.76	0.69
	Mean	0.75	0.72	0.75
	p	vs. MRI 0.98	vs. CT 0.18	vs. PET 0.17
MDG	2	0.60	0.55	0.63
	3	0.22	0.25	0.73
	5	0.42	0.72	0.66
	8	0.11	0.24	0.38
	10	0.00	0.06	0.22
	12	0.42	0.71	0.66
	13	0.62	0.58	0.64
	Mean	0.36	0.47	0.58
	p	vs. MRI <0.001	vs. CT <0.001	vs. PET <0.001

*DSC, Dice Similarity Coefficient; LDG, Less Difficult Group; MDG, More Difficult Group*.

## Discussion

To the best of our knowledge, this is one of the first study showing that the addition of chest MRI to CT and PET reduces inter-observer variability by decreasing variations in volume dimensions and increasing overlapping between contours. Moreover, its addition decreased the volume of GTV compared to volumes obtained after PET/CT aid.

PET/CT is considered the reference imaging modality to aid in delineation for lung tumors (22). Nevertheless, it presents some limitations in precise morphological delineation due to poor spatial resolution and partial volume effect (8). The study showed that accuracy was enhanced further with every addition of a new imaging modality, even if it was not statistically significant for PET/CT compared to CT alone (*p* = 0.056). Despite the fact that inter-observer variability decreased after the addition of PET/CT compared to CT alone, it decreased even more after the addition of MRI (COV 0.58 vs. 0.53 vs. 0.38, PET vs. MRI *p* = 0.06; DSC 0.56 vs. 0.60 vs. 0.67, PET vs. MRI *p* < 0.001). The MRI phase was not statistically superior to PET phase in COV, but the analysis was performed on only 14 values, showing a possible trend of improvement in the homogeneity of volume sizes. The absence of significant difference between PET/CT and CT alone may be the consequence of the patients' selection criteria, for which PET/CT was not enough to delineate the tumor. In the group of more difficult cases, the COV was increased (0.67 vs. 0.32; *p* < 0.001) compared to the group of less difficult cases, while the DSC was decreased (0.47 vs. 0.74; *p* < 0.001), expressing more variability for more difficult cases. This indicates logical concordance between assessment of case difficulty and index analysis (COV and DSC) with more heterogeneous volumes in difficult cases. In the more difficult group, both COV and DSC varied between phases much more significantly than in the less difficult group, where neither PET/CT nor MRI improved them. Karki et al. published in 2017 a similar study (23). Seven observers (five radiation oncologists and two radiologists) had to delineate the GTV of lung tumors and their associated nodes for 10 patients. There was also three phases of delineation; however the MRI phase was delineated on MRI and not on CT with the help of MRI as in our study. In this study, volumes obtained after the addition of MRI were smaller than those obtained with PET/CT (mean volume 31.7 vs. 27.7 cm^3^; median 6.4 vs. 4.8 cm^3^; *p* = 0.015). Fleckenstein et al. (24) compared the GTV contoured on CT for 16 lung tumors, with the help of PET/CT when necessary, to the GTV obtained from diffusion weighted sequences. The anatomical plausibility was checked using T2-weighted sequences. An acceptable level of agreement was observed with a mean DSC of 0.67 between the two contours. Oppositely to our study, their volumes were modestly larger for the diffusion-weighted GTV. In our study, T1- and T2-weighted sequences were used for delineation by the observers. This may explain the difference in results between the two studies, especially knowing the inherent spatial distortion of the diffusion-weighted sequence (25). The lower spatial resolution of the diffusion-weighted sequence favors the use of a morphological sequence to precisely delineate the tumor limits.

The CT phase, without PET or MRI, allowed good calibration of the different phases of delineation and more precise assessment of the input of each modality. Despite the fact that observers in this study did not have much experience in chest MRI as it is an imaging modality not performed in daily routine for each patient and were not helped by a radiologist, the use of lung MRI reduced inter-observer variability. Although radiologists and radiation oncologists cooperate easily if necessary, the help of a radiologist was not permitted during this study to avoid an artificial decrease in inter-observer variability. Further training of the physicians in chest MRI interpretation and the help of a radiologist would theoretically further improve contour quality and inter-observer variability. Moreover, our study corresponds to daily practice with both senior and resident being observers. In addition, all MRI sequences were registered together with shifts due to the different time acquisitions and the respiratory trigger technique. The rigid registration of the MRI with the planning CT was done for a single sequence (T1-weighted sequence). Therefore, the shifts observed between the sequence registered with the planning CT and the other sequences were observed between these other sequences and the planning CT. Thus, observers could not benefit completely from the use of every sequence to help in delineation. The registration of the planning CT and the PET/CT has been realized between the planning CT and the CT of PET/CT. Thus, the problem is similar with PET/CT, as spatial misregistration does occur between the PET and the CT of the PET/CT due to different moments and duration of acquisition (26). No reference contour was considered in our study since it is difficult to define one in cases of discrepancies between imaging modalities, none of which are considered a gold standard. Ideally, contours should be compared to pathological data, such as that already done for PET/CT (27), but this requires prospective studies on operable patients. Therefore, in our study, improved consistency in GTV delineation was shown with the help of MRI without any certainty of its increased accuracy.

Our study presents several limits. It is a monocentric study. Also, we did not assess ITV or PTV as the first step in delineation is to define a GTV. These volumes (ITV and PTV) have variable definition depending on techniques, tumor location and department habits. Thus, by focusing on the GTV, our results can be applied to all radiotherapy techniques such as intensity modulated radiation therapy, stereotactic body radiation therapy, and even proton therapy. Some patients did not have the same position during the different imaging modalities and planning CT, but we think that it did not impact significantly the results as the registration was mainly based on the target volume. We did not analyze inter-observer variability for anatomic regions, however most of our cases had similar difficulties because of atelectasis and peri-mediastinal location. And finally, the few number of clinical cases could be regarded as a limitation.

Chest MRI presents several advantages. It offers diverse information from various sequences. In our study, the sequences more clearly showing the tumor varied according to the considered cases. In addition, it does not expose patients to ionizing radiation. Because of its higher contrast and spatial resolution, chest MRI can be useful for tumor delineation. It can also be helpful in particular cases. For example, case three presented hypermetabolism of the tumor and atelectasis at PET/CT, making the distinction between tumor and atelectasis difficult; however, it was easily delineated on MRI (Figure 3). Moreover, one main perspective is the increasing use of PET combined with MRI since 2010 (28). The simultaneous acquisition of PET emission scan data and multiple MRI sequences reduces acquisition time. PET/MRI was comparable to PET/CT for accuracy of TNM staging and characterization of pulmonary lesions (28, 29). It has still not been evaluated for delineation but our study shows a potential interest for this imaging modality, which combines the advantages of PET and MRI with minimized misregistration (28, 29). A potential further step is the analysis of chest MRI influence on internal target volume for stereotactic radiotherapy. 4D PET/CT has been compared to 3D PET/CT and has shown increased inter-observer agreement in defining the internal target volume of centrally located lung tumors (30).

## Conclusion

The adjunction of chest MRI to multimodal (CT and PET/CT) delineation yielded a significant decrease in inter-observer variability delineation for poorly defined GTV. We suggest the use of chest MRI as an aid for lung tumor delineation in cases of associated atelectasis, iodine contrast media contraindication associated with peri-hilar tumors, recurrent disease or residual tumor within radiation pneumonitis, and radiological volume unconformity with CT and PET/CT as a problem-solving tool that should be explored in further prospective studies.

## Data Availability

The datasets generated and/or analyzed during the current study are available from the corresponding author on reasonable request.

## Ethics Statement

All patients had been informed about the opportunity of using the recorded data in later studies following French law on informatics and liberty and local ethic committee approved the study.

## Author Contributions

LB, HJ, and XM designed the study. LB, HJ, AE, and TL verified the analytical method. LB and HJ selected the cases. LB organized the contouring phases. AE, AC, DP, RD, and XM performed the contouring phases. LB did the statistical analysis. LB, HJ, AE, TL, EL, and XM wrote the manuscript. All authors have read and approved the final version of the manuscript.

### Conflict of Interest Statement

The authors declare that the research was conducted in the absence of any commercial or financial relationships that could be construed as a potential conflict of interest.
